# Understanding How Domestic Violence Support Services Promote Survivor Well-being: A Conceptual Model

**DOI:** 10.1007/s10896-017-9931-6

**Published:** 2017-07-18

**Authors:** Cris M. Sullivan

**Affiliations:** Psychology Department, Michigan State University, 316 Physics Rd., E. Lansing, MI 48824 USA

**Keywords:** Intimate partner violence, theory of change, conceptual model, domestic violence agency

## Abstract

Domestic violence (DV) victim service programs have been increasingly expected by legislators and funders to demonstrate that they are making a significant difference in the lives of those using their services. Alongside this expectation, they are being asked to describe the Theory of Change guiding how they believe their practices lead to positive results for survivors and their children. Having a widely accepted conceptual model is not just potentially useful to funders and policy makers as they help shape policy and practice -- it can also help programs continually reflect upon and improve their work. This paper describes the iterative and collaborative process undertaken to generate a conceptual model describing how DV victim services are expected to improve survivors’ lives. The *Social and Emotional Well-Being Framework* guiding the model is an ideal structure to use to describe the goals and practices of DV programs because this framework: (1) accurately represents DV programs’ goal of helping survivors and their children thrive; and (2) recognizes the importance of community, social, and societal context in influencing individuals’ social and emotional well-being. The model was designed to guide practice and to generate new questions for research and evaluation that address individual, community, and systems factors that promote or hinder survivor safety and well-being.

Domestic violence^1^ (DV) is a serious and pervasive social problem with devastating physical, psychological, and economic consequences for victims. Over one-third of women, and one in four men, in the United States have been physically assaulted, sexually assaulted, and/or stalked by an intimate partner (Black et al. 2011). In addition to physical health consequences from abuse, DV has been found to relate to Post Traumatic Stress Disorder (PTSD), depression, and suicide ideation (Coker et al. 2002; Pico-Alfonso et al. 2006; Zlotnick et al. 2006). In order to cope, some survivors turn to alcohol or other drugs (Fowler and Faulkner 2011; Martino et al. 2005). Finally, DV often includes economic abuse, including preventing survivors from working or going to school, sabotaging their employment or housing, or ruining their credit (Adams et al. 2008; Alexander 2011). These tactics can lead to job loss, homelessness, and financial ruin.

Given the pervasiveness of DV and its deleterious impacts, communities throughout the United States have created a broad range of supports for survivors and their families, including shelter programs, advocacy services, transitional housing, support groups, supervised visitation centers, outreach, and counseling services. These are often nonprofit organizations, relying on volunteers as well as paid staff, trying to meet needs that are often overwhelming. In their most recent national census of DV programs, the National Network to End Domestic Violence reported that there were over 12,000 unmet requests for services across the country in just one day (National Network to End Domestic Violence 2016).

## The Need for a Conceptual Model Describing How DV Programs Achieve Change

DV programs have been increasingly expected by state and federal funders to demonstrate that they are making a significant difference in the lives of those using their services (Macy et al. 2009; Sullivan et al. 2008). Alongside this expectation, they are being asked to describe their theory of change, or conceptual model, justifying why they engage in the practices they do. Having a widely accepted conceptual model can be useful to a number of constituents: it can help grantors allocate limited funding in ways they are confident will lead to success, and it can assist policy makers to design local, state and federal policies that are most likely to reduce domestic violence and assist survivors. Equally important, a conceptual model can help DV programs continually examine their own accountability: How well is a program meeting its goals? Is a program engaging in practices that are likely to lead to their desired goals? Should staff^2^ be doing anything differently? This paper describes the collaborative and iterative process undertaken to generate a conceptual model describing how DV services are expected to lead to positive changes in survivors’ lives.

## Method

To initially draft a conceptual model for DV services, the author drew on her then 15 years experience helping local DV programs across numerous states critically think about and evaluate their services. A central part of this early work had involved asking advocates from around the country what they hoped their programs would accomplish. While the immediate answer to this question was often to “end domestic violence,” in-depth conversations about why DV happens, who is responsible for it, and what victim service programs can realistically hope to accomplish, led to more carefully considered responses. Consensus was quickly reached that, while the goal of the nationwide movement (which includes state coalitions, national resource centers, batterer intervention programs, and other efforts) is to end DV, the role of victim service agencies is to promote survivors’ and their families’ well-being. It is not enough that a survivor be physically safe from their abuser if that safety comes at the cost of the survivor’s sovereignty and health. Therefore, safety was not viewed as the ultimate outcome of victim service programs. Further, advocates and survivors had stressed that psychological well-being was also an insufficient objective -- and put too much emphasis on changing the survivor rather than social conditions. As a result of these numerous conversations, *social and emotional well-being* was agreed upon by advocates across numerous and geographically diverse states as the ultimate goal of DV victim service programs.

Further drawing on earlier evaluation efforts with local programs and state coalitions, it was decided not to describe the efforts of DV programs by particular service categories, such as support groups, legal advocacy, shelter, or transitional housing, for two reasons. First, many activities cross service categories (e.g., safety planning, information sharing). Second, as staff seek to broaden their work over time they do not want to be limited by focusing only on current commonly offered services. Therefore, the conceptual model delineates the types of activities staff engage in that are expected to lead to desired change, regardless of how and where those activities occur.

The next step in the process was to review the empirical evidence behind (1) the factors that promote social and emotional well-being, and (2) the known impact of DV programs on survivors’ lives. This evidence was used to further refine the model and to more clearly delineate the pathways through which change was expected to occur.

Throughout the process of the conceptual model being developed, the National Resource Center on Domestic Violence hosted three meetings of national experts to gain further input. Experts included representatives from the Family Violence Prevention and Services Program at the Department of Health and Human Services (DHHS), the ten (at the time) DHHS-funded members of the Domestic Violence Resource Network, state coalitions and local DV programs, as well as researchers, FVPSA/STOP state administrators and other national partners. Expert advisors provided feedback on multiple iterations of the model over time, and after being finalized in 2011, it was disseminated nationally through webinars, in-person workshops, and the DV Evidence Project website (http://www.dvevidenceproject.org). Each dissemination activity involved explaining to participants that this was a work in progress and that their honest and critical feedback was important in order to modify it accordingly. Those working with marginalized communities were especially targeted for feedback about the model’s generalizability. Feedback has been uniformly positive, with advocates across a variety of settings and states agreeing that the model represents their work and goals. The model has also been adopted, with minor modifications, across Ireland (http://www.safeireland.ie/wp-content/uploads/A-Framework-Domestic-Violence-Service-Provision-Women-Children-IRL.pdf).

## Conceptual Model: How DV Programs Promote Survivor Well-Being

### Theory Guiding the Work of DV Programs

A theoretical framework that accurately describes how DV program services are expected to lead to positive outcomes is *Conservation of Resources (COR)* theory*.* This theory postulates that psychological distress following traumatic or highly stressful life events is strongly influenced by “resource loss,” in that trauma often results in individuals losing economic, social, and interpersonal resources central to their well-being (Hobfoll 1989, 1998, 2001). For DV survivors, this can include consequences such as having to relocate and leave family and friends, in addition to experiencing physical injuries, depression, and/or a reduced sense of self. The theory posits that if this trauma-induced ‘resource loss’ is followed by resource *gain*, psychological distress will be reduced and well-being will be increased. For example, if safety is re-established, justice is achieved, and skills are enhanced, these resource gains would counteract the resource losses and reduce the negative impact of the trauma.

Hobfoll (2001) also refers to resource loss and gain “spirals,” explaining that resource loss often results in further resource loss, while gain often initiates further gain. This theory was supported for DV survivors by the work of Sullivan and colleagues (Anderson et al. 2003; Bybee and Sullivan 2002; Sullivan and Bybee 1999; Sullivan et al. 2002), who interviewed women with abusive partners across two years after they had exited a DV shelter. Half of the sample had been randomly assigned to receive intensive advocacy services designed to increase their access to community resources and social support post-shelter. Consistent with COR theory, women who had worked with advocates for 10 weeks continued to show improvement even two years later compared to women in the control condition. They reported more social support, greater effectiveness accessing resources, higher quality of life, and lower reabuse. COR theory was further supported through a longitudinal study conducted by DePrince et al. (2012), which found that DV survivors who did *not* receive proactive advocacy experienced increased distress over time (resource loss spiral). There has been considerable empirical support for this theory across numerous other populations (e.g., Hobfoll et al. 2011, 2006).

### The Positive Changes that DV Programs Support Survivors to Achieve

Given the intentionally individualized nature of DV programs’ work with survivors (Sullivan et al. 2008), the ultimate goal that programs are working toward can be described as enhancing survivors’ and their children’s subjective well-being, or quality of life (Diener 2009). Subjective well-being has been conceptualized as including three components: (1) a cognitive appraisal that life is good [life satisfaction]; (2) experiencing positive levels of pleasant emotions; and (3) experiencing relatively low levels of negative moods. *Social* well-being includes the extent to which one has the material and interpersonal resources needed to be healthy, safe, and happy.

There is considerable empirical evidence describing how social and emotional well-being is impacted by intrapersonal, interpersonal, and social factors. At the intrapersonal level, well-being is influenced by two factors that are often damaged through DV victimization: self-efficacy and hopefulness.

Self-efficacy is the belief that one is competent and able to perform the actions needed to achieve goals important to them (Bandura 1977). Across many studies and numerous populations, self-efficacy has been found to influence one’s social, physical and emotional well-being (Boehmer et al. 2007; Hack and Degner 2004; Hampton 2004; Hochhausen et al. 2007). A DV survivor’s self-efficacy is often diminished not only by the abuser’s pattern of ridicule, control and domination, but also by prior community responses that have not only failed to help but that may have been revictimizing or made the situation worse (Campbell 2006, 2008; Rivera et al. 2012).

DV staff recognize that self-determination and agency are socially situated. Both are influenced strongly by a person’s history, current and past interpersonal relationships, social location, and community and cultural context. Self-determination does not necessarily mean independence or individual autonomy, and these constructs are intentionally rejected within some cultures that highly value interdependence and communalism. Helping a survivor gain or maintain control over their decisions and actions can and does occur within multiple contextual frameworks, and in consideration of a survivor’s family, community, and cultural needs.

One’s sense of hopefulness for the future has also been identified as a strong predictor of well-being. Hope has been defined as the belief in a positive tomorrow (Hinds 1984; Snyder et al. 1997; Stoddard et al. 2011). The extent to which one feels hopeful, however, is intricately related to one’s sense that they can *create* that ‘positive tomorrow.’ As Snyder et al. (1991) noted, “hope is influenced by the perceived availability of successful pathways related to goals. The pathways component refers to a sense of being able to generate successful plans to meet goals” (pp. 570–571). Hope, then, is distinct from but interrelated with one’s sense of self-efficacy. Hope is viewed as a critical factor relating to overall well-being because it fuels one’s willingness to do what is necessary to maintain or regain health and well-being (Snyder 2002). There is strong support for this assertion. Elevated hope has been found to relate to reductions in PTSD, anxiety, and depression (Gilman et al. 2012; Larson et al. 2007; Wu 2011). Schrank et al. (2012) conducted a meta-review of studies examining the relationship between hope and well-being, and concluded that programs intending to increase hope should include components that involve both (1) staff collaborating with clients to meet client goals; and (2) an emphasis on efficacy, spirituality and well-being. Not coincidentally, these components are often central to the work of DV programs.

While these two intrapersonal factors (self-efficacy, hopefulness) are directly related to well-being, interpersonal and social factors are equally important determinants (e.g., Bonanno et al. 2010, 2007; Galea et al. 2002; Hobfoll 2001; Norris et al. 2002). Financial and housing stability, safety, community supports, and access to healthcare are examples of social factors that consistently have been found to relate to adults’ and children’s well-being (Braveman and Gruskin 2003; Ferguson 2006; Raphael 2006). Intrapersonal, interpersonal and social factors are also mutually reinforcing; as we achieve success in meeting our goals we feel more efficacious and hopeful, and this self-efficacy then leads to greater success.

### Activities Designed to Achieve These Goals

DV programs engage in a wide range of activities designed to positively impact the social and emotional well-being of both survivors and their children. Specifically, they work to (1) increase survivors’ and their children’s sense of self-efficacy as well as their hope for the future, and (2) directly increase their access to community resources, opportunities, and supports (including social support). Consistent with Conservation of Resources theory, these improvements are expected to create a positive spiral in survivors’ lives, resulting in more positive social and emotional well-being over time.

While the actual programs offered may differ across agencies (e.g., shelter, counseling, advocacy, transitional housing, supervised visitation, children’s programs, support groups), services for both survivors and their children tend to share eight key features. In partnership with the survivors and children, DV program staff engage in the following activities: (1) providing information about adult and child survivors’ rights, options and experiences; (2) safety planning; (3) building skills; (4) offering encouragement, empathy, and respect; (5) supportive counseling; (6) increasing access to community resources and opportunities; (7) increasing social support and community connections; and (8) community change and systems change work. Each is briefly described next.

1. ***Provide Information*** *.* Given that knowledge is power, a primary goal of DV programs is to increase survivors’ and their children’s knowledge about a range of issues important to their well-being. This entails informing survivors about their rights, options, and the community resources they have available to them. Staff work to provide any and all information survivors might need to understand their experiences within the larger sociopolitical context, so that they can make the most informed choices they can, given their situation.
2. ***Safety Plan.*** A basic principle of every DV program is to engage in safety planning with survivors and their children (Davies and Lyon 2013). Staff recognize that “safety plan” is a verb rather than a noun, and that strategies must be flexible and individualized to each survivor’s experience and context. While efforts on the parts of survivors may or may not be successful, given that perpetrators are ultimately responsible for their behavior, various strategies are discussed so that survivors can decide for themselves what might reduce their future risk of abuse.
3. ***Build Skills***. Knowledge is critically important, but having the skills to put knowledge into practice is crucial to enhancing self-efficacy. DV program staff use a variety of strategies, including instruction, modeling, and role playing, to help survivors and their children enhance the skills they self-identify as needing.
4. ***Offer Encouragement, Empathy and Respect.*** Across all services, DV staff are expected to treat survivors with empathy, support and respect. Staff are trained to be nonjudgmental, respectful of differences, and to be culturally competent. Behaving in this manner has been empirically linked to increases in clients’ sense of self and self-efficacy (Maton et al. 2004; Saleebey 2006). Staff members’ encouragement, empathy and respect encourage survivors to recognize their skills and strengths. Staff also address the range of physiological factors that can impact people’s ability to engage in new behaviors (Hyde et al. 2008). For example, staff might work with survivors to recognize signs of anxiety (e.g., “butterflies in the stomach,” fear, trembling), to normalize this, and to offer strategies for self-regulation.
5. ***Supportive Counseling.*** Whether through individual counseling, support groups, crisis intervention or casual conversations, staff help survivors and their children understand that they are not alone in their experience and are not responsible for their victimization. They also help them understand common responses to trauma (e.g., trouble concentrating, sleep problems, being easily startled) and provide them with the knowledge, skills and time they need to heal.
6. ***Increase Access to Community Resources and Opportunities.*** Empowerment-based advocacy involves working actively with survivors to help them obtain limited or difficult-to-access resources and opportunities. The role of the advocate is to engage in dialogue and critical analysis with the survivor, reviewing all sides of the issue and determining costs and benefits of different courses of action. Advocates also need to know relevant state and local laws and policies, and they should know individual people in frequently used agencies who are in control of needed resources.
7. ***Increase Social Support and Community Connections.*** Social support is critical to the well-being of all adults and children, and is especially important for DV survivors. Many survivors want to maintain strong ties in their communities or need to build new community networks, and program staff can be extremely helpful in honoring and supporting this need.
8. ***Community Engagement and Social Change Work.*** Recognizing that well-being is not independent from community-level factors, staff do not focus solely on working with individual survivors. They also engage in a variety of efforts to create communities that hold those who use violence accountable, promote justice, and that provide adequate resources and opportunities for all community members. This is accomplished through systems-level advocacy efforts (generally targeted at the criminal justice, health care, welfare, child protective service, and other systems), prevention activities, community education activities, and collaborative community actions.

The goal of most, if not all, DV programs is to help create communities that value all of their members and that promote individual and community well-being. This work involves a great deal of time and energy on the part of DV program staff, who often engage in their communities at multiple levels. They likely participate in Coordinated Community Response (CCR) Councils, meet regularly with key community members to improve protocols, policies, and practices, work on related social issues (such as poverty, discrimination, housing, employment, child welfare), and engage in cross-trainings with other professions. These complex, time-consuming, and generally underfunded efforts are key to DV programs’ social change work.

**Fig. 1:**
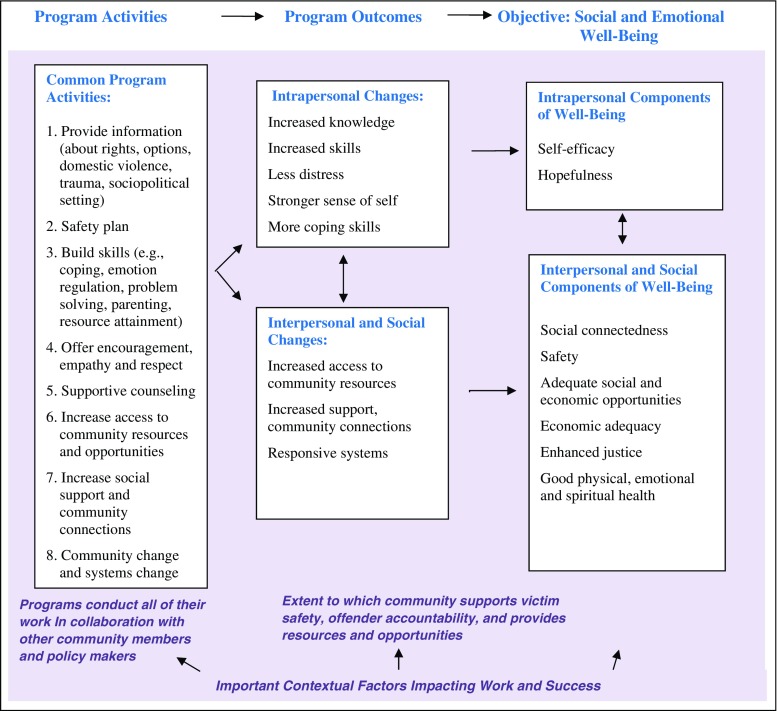
Conceptual Model Illustrating How Domestic Violence Program Activities Impact Adult and Child Survivors’ Well-Being

DV advocates often refer to the underlying philosophy guiding these eight components of their work as engaging in “empowering practice.” Empowering practice involves interacting with survivors in ways that increase their power in personal, interpersonal and political arenas (Cattaneo and Goodman 2015; Goodman and Epstein 2008; McGirr and Sullivan 2017). It is a helping relationship through which the staff member shares power with the survivor, and is a facilitator, not a director, of services. Direct outcomes of these program activities can be documented at intrapersonal, interpersonal and social levels. Intrapersonal changes include both cognitive (e.g., increased knowledge and skills) and emotional (e.g., feeling more hopeful) improvement. Interpersonal changes would include such things as increased safety and social support, while social-level changes might include increased access to community resources. Figure 1 illustrates the conceptual model describing how program service components are expected to impact the factors that influence well-being.

### Evidence Linking DV Program Activities to Desired Outcomes

Although few in number, the studies that have evaluated the most commonly offered services (e.g., shelter, advocacy, support groups, counseling) provided by nonprofit DV organizations suggest that these programs do indeed positively impact numerous factors predictive of well-being. Survivors who have used shelters, for example, report feeling safer, more hopeful, and having more safety strategies as a result of their shelter stay (Chanmugam 2011; Few 2005; Goodkind et al. 2004; Itzhaky and Porat 2005; Sullivan and Virden 2017a, b; Tutty 2006; Tutty et al. 1999; Wettersten et al. 2004). Advocacy services have been shown to lead to survivors experiencing less violence over time, less difficulty accessing community resources, increased social support, and higher quality of life (Allen et al. 2004; Bybee and Sullivan 2002; Sullivan and Bybee 1999). Support groups have led to survivors feeling a greater sense of belonging and higher self esteem, while experiencing less distress (Constantino et al. 2005; Tutty et al. 1993). A group focused on survivors’ mothering increased women’s parental self-efficacy and optimism about the future while decreasing their mothering-related stress (Peled et al. 2010). Counseling has led to decreases in depression, anxiety and PTSD symptoms, while helping survivors feel better about their lives (see Warshaw et al. 2013 for a review). Therapeutic interventions for children have been shown to improve their self concepts and reduce their behavioral problems (Graham-Bermann et al. 2007; Smith and Landreth 2003; Sullivan et al. 2002; Tyndall-Lind et al. 2001).

Although the aforementioned studies provide promising evidence for the commonly offered services of many DV programs, many have been critiqued for their serious methodological shortcomings, including lack of comparison or control groups, small sample sizes, reliance on self-report data, and insufficient inclusion of diverse clients (see Rivas et al. 2015; Sullivan 2005 for critiques). While the evidence for the effectiveness of advocacy is robust (Rivas et al. 2015) and evidence for counseling services provided by licensed therapists is strong (Stover et al. 2009), a great deal more methodologically rigorous research is needed to examine what works, for whom, and under what circumstances.

### Conclusions

DV programs work not only to protect survivors and their children from further harm, but to promote their long-term social and emotional well-being. The *Social & Emotional Well-being Framework*, therefore, reflects the mission of DV programs, and provides a useful model for organizing and articulating how the work of these programs promotes the well-being of survivors and their children over time.

Consistent with Conservation of Resources theory, DV programs try to repair the ‘resource loss’ that generally follows traumatic events and to engage with survivors and their children to instigate more ‘resource gains.’ They do this by enhancing survivors’ and their children’s knowledge, skills, self concepts, sense of hope, social connections, safety, health, stability, and access to community resources. They also work closely with community members and systems to create more broad-based change for survivors and their families. The expectation is that these improvements will create a positive spiral, resulting in more positive social and emotional well-being over time.

While this model was intentionally created as an initial step to focus on *what* DV programs do to achieve lasting change for survivors and their children, it is equally if not even more important to examine *how* program staff work with survivors. DV programs’ basic tenets include assisting survivors in ways that are empowering (Cattaneo and Goodman 2015), trauma informed (Ferencik and Ramirez-Hammond 2011; Goodman et al. 2016b), and culturally relevant (Burman et al. 2004; Lockhart and Danis 2010). The scant research that has examined the relationship between staff and survivors has found it to be important (Allen et al. 2013; Goodman et al. 2016a; Melbin et al. 2003); more research is needed to examine the relative influences of different components of DV staff efforts on achieving positive results.

In short, the empirically supported conceptual model described in this paper identifies the pathways through which DV services lead to short- and long-term positive outcomes for survivors and their children. While there have been few studies that have rigorously evaluated the impact of DV services, the evidence that does exist is promising, and suggests that these programs are engaging in effectual practices that are likely to achieve their goal of enhancing the well-being of survivors and their children (Macy et al. 2009). If local, state and federal policies that support these services are continued or strengthened, the lives of survivors and their families can only be enhanced. However, a great deal more needs to be understood, especially regarding what might work best, for whom, and under what conditions. The model is presented to spark further dialogue about, and research into, the mechanisms through which different activities can and do lead to positive outcomes for DV survivors and their children.

## Footnotes

1 The terms domestic violence and intimate partner violence are often used interchangeably to refer to physical, psychological, sexual and economic abuse perpetrated by a partner or ex-partner to achieve power and control in the relationship. Because service programs for victims and state coalitions use the term domestic violence over intimate partner violence, domestic violence is used throughout this paper.

2 Throughout this manuscript the word ‘staff’ refers to both paid employees and volunteers working within domestic violence programs, as most programs rely on at least some trained volunteer labor to achieve their goals.
